# Immune Responses of a Native and an Invasive Bird to Buggy Creek Virus (*Togaviridae*: *Alphavirus*) and Its Arthropod Vector, the Swallow Bug (*Oeciacus vicarius)*

**DOI:** 10.1371/journal.pone.0058045

**Published:** 2013-02-27

**Authors:** Carol A. Fassbinder-Orth, Virginia A. Barak, Charles R. Brown

**Affiliations:** 1 Biology Department, Creighton University, Omaha, Nebraska, United States of America; 2 Department of Biological Sciences, University of Tulsa, Tulsa, Oklahoma, United States of America; Blood Systems Research Institute, United States of America

## Abstract

Invasive species often display different patterns of parasite burden and virulence compared to their native counterparts. These differences may be the result of variability in host-parasite co-evolutionary relationships, the occurrence of novel host-parasite encounters, or possibly innate differences in physiological responses to infection between invasive and native hosts. Here we examine the adaptive, humoral immune responses of a resistant, native bird and a susceptible, invasive bird to an arbovirus (Buggy Creek virus; *Togaviridae*: *Alphavirus*) and its ectoparasitic arthropod vector (the swallow bug; *Oeciacus vicarius*). Swallow bugs parasitize the native, colonially nesting cliff swallow (*Petrochelidon pyrrhonota*) and the introduced house sparrow (*Passer domesticus*) that occupies nests in cliff swallow colonies. We measured levels of BCRV-specific and swallow bug-specific IgY levels before nesting (prior to swallow bug exposure) and after nesting (after swallow bug exposure) in house sparrows and cliff swallows in western Nebraska. Levels of BCRV-specific IgY increased significantly following nesting in the house sparrow but not in the cliff swallow. Additionally, house sparrows displayed consistently higher levels of swallow bug-specific antibodies both before and after nesting compared to cliff swallows. The higher levels of BCRV and swallow bug specific antibodies detected in house sparrows may be reflective of significant differences in both antiviral and anti-ectoparasite immune responses that exist between these two avian species. To our knowledge, this is the first study to compare the macro- and microparasite-specific immune responses of an invasive and a native avian host exposed to the same parasites.

## Introduction

Different host species often vary considerably in their ability to immunologically resist certain parasites [1], [2]. Recently it has been suggested that one determinant of a host's susceptibility to parasites or pathogens may be whether or not a host exhibits an invasive life history [3]. Characteristics associated with success as an invasive may be either positively or negatively related to immune function [4], [5]. Consequently, the transmission dynamics of host-parasite systems can be altered in several possible ways when an invasive host colonizes an area and comes into contact with native hosts and parasites. Invasive host species may introduce a new pathogen (with which the invasive has co-evolved), causing naïve, native species to become susceptible hosts for the novel pathogen (the Novel Weapon Hypothesis [6], [7]). In these cases, the invasive host may be at an advantage over the native hosts who have had no evolutionary history with the introduced parasite. On the other hand, when an invasive species invades a new environment, it may also encounter a novel parasite and thus be a more susceptible host than those native hosts that have co-evolved with the parasite [8], [9].

One example of a natural host-pathogen system in which a non-native invasive species has recently assumed a role in the transmission cycle [9] is that of the alphavirus, Buggy Creek virus (BCRV; *Togaviridae*: *Alphavirus*), that historically was amplified only among its vertebrate host, the cliff swallow (*Petrochelidon pyrrhonota*; [10]–[12]). This virus is restricted to a unique ecological setting in which it is transmitted solely by its vector, the swallow bug (Hemiptera: Cimicidae: *Oeciacus vicarius*), to the cliff swallow, the primary host for the ectoparasitic bug [13], [14]. Following the introduction of European house sparrows (*Passer domesticus*) to North America in the 1800's [15] and their subsequent invasion of cliff swallow colonies where they usurp the swallows' nests [16], swallow bugs have switched to sparrows as alternative hosts in some cases, and in the process the bugs have exposed house sparrows to BCRV, a novel pathogen [17], [18].

The invasion of house sparrows into swallow colonies provides a number of opportunities to study how this invasive species may differ from the native species (cliff swallow) in its immune responses to both the virus and the ectoparasitic bugs. Although birds are the principal reservoirs for several alphaviruses of medical importance (e.g. western equine encephalomyelitis, eastern equine encephalomyelitis, Sindbis virus [19]), relatively little is known about avian immune responses to any of the alphaviruses or the factors causing variability in host susceptibility to either BCRV or, more generally, other arthropod-borne viruses (arboviruses) such as West Nile virus. Furthermore, ectoparasites such as the hematophagous swallow bugs can exert strong selection pressures on their avian hosts by decreasing survival and future reproduction [20]–[23] and presumably also affecting evolution of the hosts' immune response. The effect of ectoparasitic arthropods on disease etiology has been studied in some mammals [24]–[26], but little is known for avian species. In particular, we know nothing about how immune responses to the same arthropod vector may vary between an invasive and a native avian species.

In this study we compare the adaptive, humoral immune responses of two bird species – the native cliff swallow and the non-native house sparrow – to the alphavirus BCRV and to the virus's ectoparasitic arthropod vector, the swallow bug. We recognize that the evolutionary histories of cliff swallows and house sparrows are different and this may account in part for observed differences between them; however, these species are both passerine birds, and passerines of different species can show similar immune responses to arthropod-borne viruses [27]. Two opposite predictions are possible. One is that due to their long co-evolutionary history with both swallow bugs and BCRV (and their heavy exposure to swallow bugs [21], [28]), cliff swallows should exhibit less strong responses (greater tolerance [29], [30]) to both the ectoparasite and the virus than should the immunologically naïve invasive house sparrow. The alternative prediction is that because invasive species sometimes invest less in their immune systems than non-invasives [3], house sparrows should show more muted responses to the ectoparasite and the virus than would the cliff swallow. We use these results to gain insight into (*i*) the potential impact of the invasive house sparrow on this host-parasite-pathogen system and (*ii*) how birds respond immunologically to alphaviruses and arthropods more generally.

## Methods

### Ethics Statement

All procedures involving animals were approved by the Creighton University Institutional Animal Care and Use Committee under protocol 0915. Blood sampling of cliff swallows was approved under the Federal bird banding permit 20948, and Nebraska Game and Parks Scientific Permit no. 254. House sparrows are a non-native species, and federal and state permits are not required for sample collection from this species.

### Study Organisms

House sparrows were introduced into North America from Europe in the mid 1800's [31], and have been present in Nebraska since about 1900 [15]. They are a widely distributed, semi-colonial species that often form aggregations of 2 to 20 nests in close proximity. They are sedentary, remaining at or near breeding sites year-round. House sparrows are multi-brooded, with nesting in our study area beginning in late April and ending in late July, with peak egg laying periods in mid May, late June, and late July. Nestlings fledge after about 14–18 days, and new broods are started soon after earlier ones fail or fledge. Cliff swallows are highly colonial, migratory passerines that breed across much of western North America [32]. They build gourd-shaped mud nests on the sides of cliff faces, inside highway and railroad culverts, and underneath bridges. In our study area, swallow colony sizes range from 2 to 6000 active nests. The mud nests persist from year-to-year and are frequently repaired and reused by cliff swallows for multiple seasons. Swallows arrive in our study area in early to mid May and typically raise a single brood, with most nestlings fledging by mid July. Individual colonies are highly synchronous and are quickly vacated by swallows after the nestlings fledge. Nestlings are in the nest for about 26 days before fledging.

During the 1960's, the construction of the interstate highway system provided alternative substrates (culverts, bridges) for cliff swallow nesting, resulting in cliff swallows' moving into peri-domestic settings and coming into contact with house sparrows. In our study area, most house sparrows have been using cliff swallow colonies for only about 30–40 years [16]. The swallow bug is a hematophagous nest ectoparasite of the cliff swallow that is not known to occur outside swallow colonies, and thus it was historically restricted to cliff swallows as hosts. The bugs take blood meals from cliff swallows and more recently, house sparrows [10], [33]. Swallow bugs disperse between nests within a colony by crawling on the substrate and disperse between colony sites by clinging to the feet/legs of cliff swallows that move from one site to another [34]. Sparrows are not known to move bugs between sites. The density of bugs in cliff swallow colonies can be quite high, with as many as 2600 bugs per cliff swallow nest and 2400 per house sparrow nest. In our study area approximately 25% of bug pools are infected with BCRV [35], [36], a single-stranded, positive-sense RNA virus antigenically and phylogenetically placed within the WEEV complex of alphaviruses [10]–[12], [37], [38]. BCRV is unusual among alphaviruses because it is vectored primarily by the ectoparasitic swallow bug, rather than by mosquitoes.

### Sample Collection

The primary study site is near the Cedar Point Biological Station (CPBS; 41°12.591′ N, 101°38.969′ W) in Keith County, Nebraska, USA, and has been previously described by Brown and Brown [28]. During the spring and summer of 2011, five cliff swallow colonies situated under highway culverts and bridges were monitored for cliff swallow and house sparrow activity to determine the effects of seasonal nesting activity on BCRV-specific and swallow bug specific antibody levels in both species (Table 1). House sparrows also use cliff swallow colonies as year-round roosting sites, and resident sparrows were sampled in late March 2011 (about 4 weeks in advance of the breeding season) to represent a pre-exposure period before swallow bugs had terminated their winter dormancy and began taking avian blood meals. Post-nesting blood samples were collected from house sparrows at colony sites in September following late nesting (that can extend into August [39]). Pre-nesting samples were collected from cliff swallows in mid-May, immediately after their return to the study area from their South American wintering grounds [28]. Post-nesting samples from cliff swallows were collected in mid-July, immediately preceding their migratory exodus from the study area (Table 1).

**Table 1:** Sampling Details.

Species	Collection Site	Collection Date	Breeding Period	*n*
House sparrow	CPBS^3^	26 March 2011	Pre-nesting	25
	CPBS	24 September 2011	Post-nesting	20
	Council Bluffs, IA (Agricultural facility)	2 September 2011	N/A	18
Cliff swallow	CPBS	26 May 2011	Pre-nesting	18
	CPBS	13 July 2011	Post-nesting	20

3 Cedar Point Biological Station, Keith County, Nebraska.

Samples were also collected from house sparrows at an agricultural facility near Council Bluffs, Pottawattamie County, Iowa, USA, with no known cliff swallow colonies within a 15-km radius of the facility. Given that swallow bugs are uniquely associated with cliff swallow colonies, and that house sparrows are a largely sedentary species [40], it is unlikely that the house sparrows in this control group had ever been exposed to swallow bugs or BCRV and thus the Iowa birds served as unexposed negative controls for both swallow bug and BCRV serological surveys.

Adult cliff swallows were captured by mist netting, and adult house sparrows were captured by mist netting and nighttime removal from nests. Following capture, birds were bled by jugular venipuncture, 100 μl of blood were collected, and sera samples were separated and stored for later use in enzyme-linked immunosorbent assays (ELISAs). Following venipuncture, pressure was applied to the collection site to prevent hematoma formation. The collection site was then inspected to ensure the cessation of any residual bleeding and birds released back to their natural surroundings.

### Antigen Production

#### Swallow Bug Homogenate

Swallow bugs were collected from nests at a cliff swallow colony near CPBS in July 2011. The nests were broken apart, and bugs were removed using forceps. Bugs were divided into pools of 10 bugs each and homogenized with 30 μl phosphate buffered saline (PBS) per bug. Homogenates were centrifuged at 10,000×*g* for 1 minute, and the supernatant was collected and filtered with a 0.2 μm filter. The protein concentration of the homogenate was determined using a Nanodrop spectrophotometer. The extract was diluted to 1 mg/mL using sterile PBS and stored at −80°C.

Viral RNA extraction from the bug homogenates was performed using the QIAmp Viral RNA Mini Kit (Qiagen, Valencia, CA). Negative and positive controls were included in each extraction procedure. Reverse transcription polymerase chain reaction (RT-PCR) was performed using the OneStep RT-PCR Kit (Qiagen, Valencia, CA), following the manufacturer's protocol to identify BCRV positive bug pools. Primers and thermocycler conditions were those of Moore et al. [36]. Amplification products were electrophoresed on a 2% UltraPure Agarose gel (Invitrogen, Carlsbad, CA). Four homogenate pools were found to be negative for BCRV (data not shown) and were combined for use in the ELISA.

#### Buggy Creek Virus Antigen

BCRV was cultured from whole blood samples diluted in BA-1 diluent according to O'Brien and Brown [17]. Briefly, Vero cells were grown in 25 cm^2^ flasks in complete growth medium (EMEM with 10% heat inactivated FBS and 1% antibiotic/antimycotic). The virus was passaged twice and 200 μl of the second passage was used to infect two additional flasks of Vero cells. Infected flasks were incubated until 50–75% cytopathic effect was observed (approximately 2–3 days), then flasks were frozen overnight at −80°C. The cells were thawed on ice, centrifuged at 1700×*g* (4°C) for 20 minutes, and the supernatant and cellular fraction were separated. The cellular fraction was re-suspended in 1.5 mL PBS and refrozen at −80°C overnight. Samples were then thawed and 1.5 mL of 0.2 M glycine (9.5 pH) were added to each tube. The cells were homogenized with a sterile homogenizer tip on an Omni Homogenizer and placed in a 37°C water bath for 4.5 hours, vortexing every hour. Levels of virus were quantified by performing a plaque assay according to Moore et al. [36]. Briefly, we added 100 μl of the viral stock supernatant to a confluent monolayer of Vero cells in 6 well plates, incubated the plates for 1 hr at 37°C, 5% CO_2_, then overlaid the monolayer with an agar overlay for plaque visualization. Plaques were scored 3–4 days later, and the final concentration of BCRV stock solution was 7.8×10^5^ PFU/mL.

The BCRV stock solution was inactivated for use in the ELISA. To do this, 3 mL of PBS with 0.5% Triton X were added, and the mixture was incubated at 4°C for 2 hours, with vortexing every half hour. It was then centrifuged at 10,000×*g* for 10 minutes at 4°C. The supernatant was frozen at −80°C for later use in the ELISA.

### ELISAs

**Figure 1:**
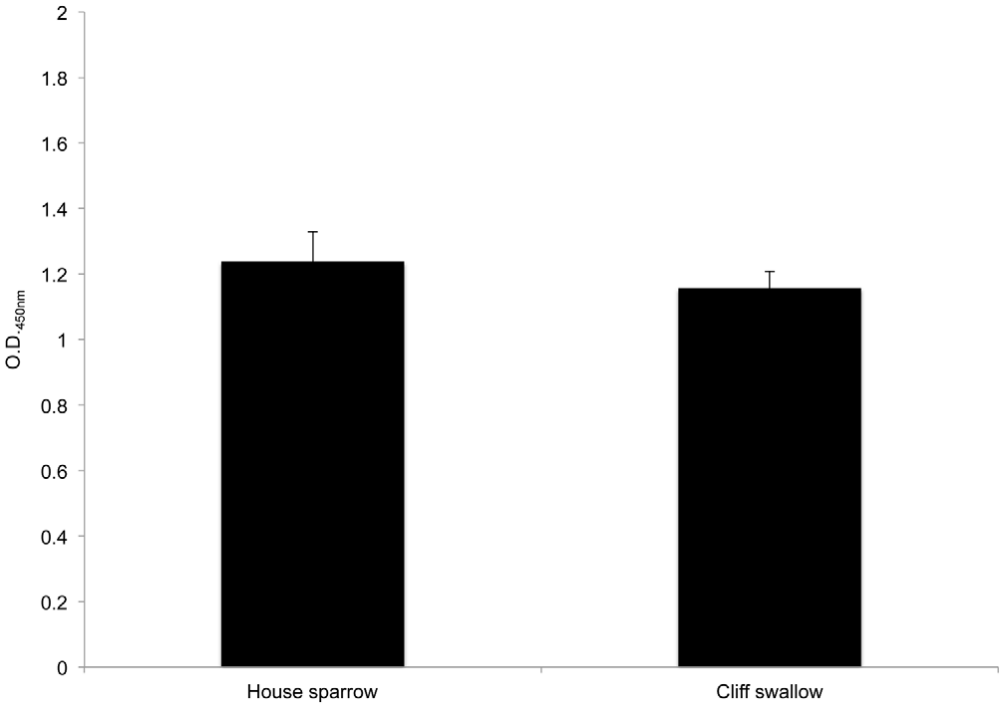
Total IgY ELISA Results. Values shown represent mean (+ SEM) optical density values (O.D._450nm_) for total IgY in cliff swallows and house sparrows. NS =  non-significant difference (*P*>0.45).

A direct ELISA was performed to determine total IgY in house sparrows and cliff swallows. A flat-bottomed 96-well plate (Nunc, Roskilde, Denmark) was coated with 100 μl of pooled house sparrow and cliff swallow sera (n = 15 individuals/species/pool) per well, diluted to 1∶100 in coating buffer (0.015 M Na_2_CO_3_, 0.035 M NaHCO_3_, pH 9.6). The plate was incubated overnight at 37°C. The coating solution was removed, 200 μl of blocking buffer (PBS with 5% non-fat dry milk, 0.05% Tween) were added to each well, and incubated at room temperature for 30 minutes. The plate was washed four times with wash buffer (PBS with 0.05% Tween) using a BioTek ELx50 Automated Strip Washer (Biotek Instruments, Winooski, VT). Fifty μl of the detecting conjugate goat anti-bird IgY-HRP (Bethyl Laboratories, Inc., Montgomery, TX) were added at 1∶1000 in blocking buffer, incubated at 37°C for 1 hour, and washed. One hundred μl of tetramethylbenzidine (TMB)-peroxidase substrate (Kirkegaard & Perry Laboratories, Inc., Gaithersburg, MD) were added to each well, incubated for 5 minutes, and the reaction was stopped with 100 μl 1 M H_2_SO_4_. Optical density (OD) values were read at A_450_ using a BioTek Synergy HT automated microplate reader (Biotek Instruments, Winooski, VT). Total levels of IgY were not significantly different between species (Figure 1), indicating sufficient recognition of IgY in both avian species by the conjugate antibody.

We performed indirect ELISAs that detected swallow bug and Buggy Creek virus (BCRV)-specific IgY. One hundred μl per well of 1∶1000 swallow bug homogenate or 1∶50 BCRV in coating buffer were added and incubated overnight at 4°C. After blocking with 200 μl of blocking buffer per well and incubating for 30 minutes, 100 μl of either cliff swallow or house sparrow sera were added at 1∶100 in wash buffer and incubated at 37°C for 1 hour. Wells were washed, and 100 μl goat anti-bird IgY at 1∶1000 in blocking buffer were added and incubated at 37°C for 1 hour. Wells were again washed, then 100 μl of TMB were added and allowed to incubate for 10 minutes at room temperature. The reaction was stopped with 100 μl of 1 M H_2_SO_4_ and plates were read at A_450_.

### Statistical Analyses

Values given are mean ± 1 SEM. Total IgY, and swallow bug and BCRV reactive antibodies were tested by ANOVA (GLM in SYSTAT [41]) according to experimental group (house sparrow control, house sparrow pre- and post- nesting, cliff swallow pre- and post-nesting). Subsequent ad hoc comparisons among experimental groups were made on the adjusted least squares from the ANOVA using the Tukey honestly significant difference test. The significance level was set at *P*<0.05.

## Results

Indirect ELISAs were successfully developed to measure swallow bug and BCRV-specific IgY levels in adult house sparrows and cliff swallows before and after nesting at selected cliff swallow colony sites in western Nebraska. Specific antibody levels in birds at the primary study area were compared to samples from a BCRV and the swallow bug- unexposed control group of house sparrows.

**Figure 2:**
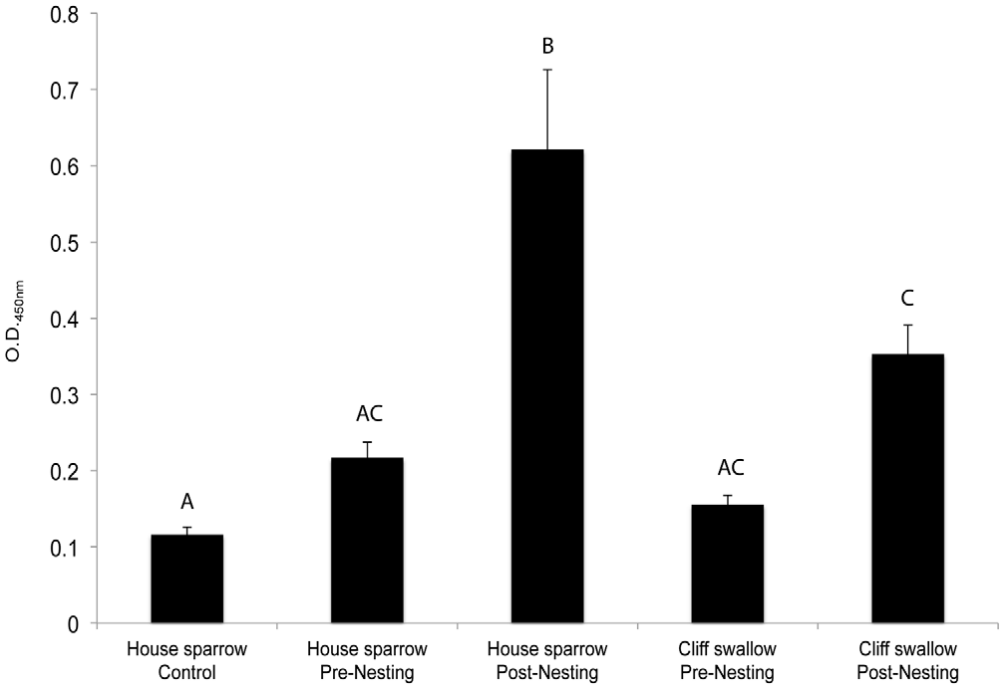
BCRV ELISA. Values shown represent mean (+ SEM) values for BCRV specific IgY levels in each group. House sparrow Control samples collected from house sparrows at Council Bluffs, Iowa (no known cliff swallow colonies in the area) in August, 2011. All other blood samples collected from house sparrows and cliff swallows at swallow colony sites in western Nebraska in 2011. Letters above bars denote statistical differences among groups (*P*<0.05); bars not sharing the same letter are different.

Seasonal variation in BCRV-specific antibodies was detected in this study (Figure 2). Pre-nesting levels of BCRV-specific antibodies in both house sparrows and cliff swallows were low and were not significantly different from the house sparrow control group (*P*>0.62). However, post-nesting BCRV-specific levels were significantly increased from pre-nesting levels in house sparrows (*P*<0.0001). In cliff swallows, post-nesting BCRV-specific antibody levels displayed a trend of significance when compared to pre-nesting levels (*P*<0.079), and were significantly higher than the house sparrow control group (*P*<0.018).

**Figure 3:**
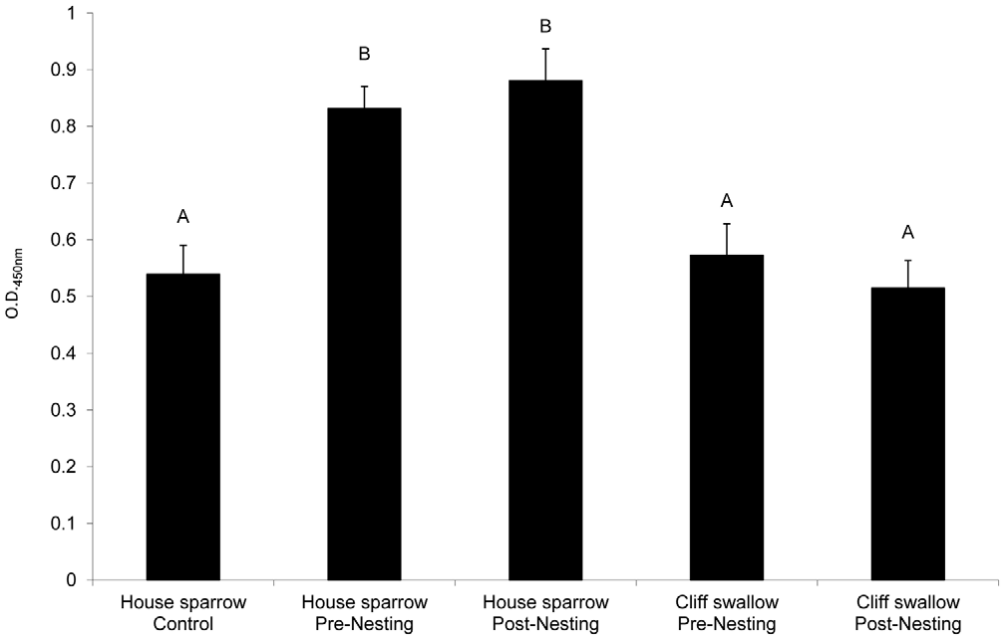
Swallow bug ELISA. Values shown represent mean (+ SEM) swallow bug-specific IgY levels in each group. Letters above bars denote statistical differences among groups (*P*<0.05); bars not sharing the same letter are different.

There were no significant effects of seasonality on swallow bug-specific antibodies in this study (Figure 3), as pre and post nesting swallow bug-specific IgY levels did not differ significantly within each species (*P*>0.99). Levels of swallow bug-specific IgY levels in cliff swallows were not significantly different from the house sparrow control group during both pre- and post-nesting periods (*P*>0.99), likely indicating that low levels of swallow bug-specific antibodies were present in adult cliff swallows in this study. However, significantly higher levels of swallow bug-specific IgY levels were found in the pre- and post-nesting house sparrow groups compared to all other groups (*P*<0.002).

## Discussion

Multiple surveys have been conducted that report on the prevalence of arboviruses or arbovirus-specific antibodies in diverse avian populations [42], [1]. However, few investigations have explored antibody levels to both arboviruses and their insect vectors in avian hosts, effects of seasonality on these antibody levels, or how invasive avian hosts may differ from native hosts in their antibody-mediated responses to viral or vector antigens. In this experiment, we detected significant differences in the antibody response patterns of house sparrows (the invasive host) compared to cliff swallows (the native host) to an alphavirus (BCRV) and its hemipteran vector (the swallow bug).

BCRV-specific antibodies varied seasonally in both house sparrows and cliff swallows, but were significantly higher in house sparrows in the post-nesting season. Seasonal variation in the prevalence of both BCRV RNA and cytopathic virus has been previously documented in swallow bugs and house sparrows [33], [43]. In swallow bugs, BCRV prevalence is significantly lower during the winter months, with the majority of the virus detected being non-cytopathic during this time [13]. Termination of diapause and commencement of blood feeding is thought to initiate cytopathic viral growth in swallow bugs [43]. The seasonal changes in BCRV in bugs have been supported by data on virus prevalence in house sparrows: the highest levels of BCRV infection in nestling house sparrows (the primary amplifiers of BCRV) occur in mid-summer, which also coincides with the time of the largest swallow bug population sizes and the highest BCRV infection levels seen during the year [14]. Previous work therefore indicates that the increase in BCRV-specific antibodies following nesting in this experiment may be explained by the increased exposure of birds to cytopathic BCRV during the nesting period, brought about in part by higher biting rates as swallows bugs increase in abundance in mid-summer [34].

The finding that house sparrows had significantly higher levels of BCRV-specific antibodies compared to cliff swallows during the post nesting period could indicate that the house sparrow is exposed to higher levels of BCRV throughout the breeding season [9], or it may indicate that the humoral immune response of these species to this arbovirus is fundamentally different, with the cliff swallow displaying a less reactive response compared to the house sparrow. Recent research in mammals suggests that neutralizing antibodies may not be required for protection against some cytopathic viral infections [44]. Therefore, although the presence of BCRV-specific antibodies in house sparrows indicates humoral immune system recognition of the virus, it may not necessarily indicate the effectiveness of the antibody-mediated response of house sparrows to clear the BCRV infection.

The relatively high background levels of swallow bug-specific antibodies detected in this experiment may have been due to the possible detection of antibodies against other hematophagous nest or feather ectoparasites that have homologous salivary components to the swallow bug, as has been shown to occur with serological assays for other insect vectors [45]. However, the significantly higher level of swallow bug-specific antibodies that were detected in the western Nebraska house sparrows compared to the control house sparrows indicates a significantly different pattern of exposure to ectoparasites between these two groups of house sparrows. Given that house sparrows are frequently exposed to swallow bugs in the Nebraska study area [9], [13], it is likely that the increase in antibody levels are reflective of an increased humoral immune response to swallow bugs in these species.

Arthropod vector-specific immune responses are found in many vertebrate populations (e.g. humans, livestock, wild birds and mammals), and salivary protein-specific antibody responses have been used as a marker for vertebrate exposure to ticks, mosquitoes, sand flies, nest flies, black flies and triatomines [46]–[52]. We detected significant levels of swallow bug specific antibodies in house sparrows but not in cliff swallows, with no evidence for seasonal variation in antibody levels in house sparrows.

The high degree of host specificity that is found in many nest-based and feather parasites suggests that co-speciation of ectoparasites and avian hosts often occurs [53]. Given that swallow bugs are restricted exclusively to cliff swallow nests and parasite infestations are often high [21], [28], co-evolution of cliff swallow and swallow bug population biology is likely [14]. One way such co-evolution may be expressed is through the ectoparasite-specific immune response of cliff swallows' evolving to be more desensitized and tolerant in nature, compared to that of the house sparrow. Desensitization may be beneficial because robust ectoparasite-specific immune responses may negatively impact the outcome of concurrent or future arbovirus infections in some vertebrate hosts, making hosts either more susceptible to infection [54], [55] or resulting in decreased levels of parasite-specific antibodies [56]. To our knowledge, no previous studies have compared the ectoparasite-specific immune responses of a historically exposed native host to a more recently exposed invasive host.

The differences in antibody-mediated immune responses to the swallow bug and BCRV observed in this experiment may help to explain the differences in BCRV susceptibility that exist between cliff swallows and house sparrows. House sparrows are more likely to be infected, exhibit higher virus titers, and develop severe BCRV-induced pathology compared to cliff swallows [9], [18]. It is plausible that the house sparrow's pattern of immune recognition of swallow bug salivary components may alter the immune response in such a way as to enhance BCRV severity in these hosts. One way this may occur is by increased cellular recruitment at bite sites, dysregulated cytokine signaling, and altered humoral immune responses, as has been seen following mosquito, sand fly, and tick bites in other vertebrate hosts [57]–[59]. More research into the mechanistic details of the immune responses of house sparrows and cliff swallows to swallow bugs and BCRV is needed to identify all immunological factors that may be involved in determining disease resistance and susceptibility in these two species.
